# A183 VARIATION IN HEALTH SERVICES UTILIZATION AND RISK OF SURGERY ACROSS CHILDREN WITH INFLAMMATORY BOWEL DISEASE: A MULTIPROVINCE COHORT STUDY

**DOI:** 10.1093/jcag/gwac036.183

**Published:** 2023-03-07

**Authors:** E Kuenzig, H Singh, A Bitton, G G Kaplan, M W Carroll, A R Otley, T A Stukel, S Spruin, A M Griffiths, D R Mack, K Jacobson, G C Nguyen, L E Targownik, W El-Matary, S Nasiri, E I Benchimol

**Affiliations:** 1 Child Health Evaluative Sciences, SickKids Research Institute; 2 SickKids Inflammatory Bowel Disease Centre, Division of Gastroenterology, Hepatology and Nutrition, The Hospital for Sick Children, Toronto; 3 University of Manitoba IBD Clinical and Research Centre; 4 Department of Internal Medicine, Max Rady College of Medicine, , University of Manitoba; 5 Research Institute at CancerCare Manitoba, Winnipeg; 6 Gastroenterology and Hepatology, McGill University Health Centre, Montreal; 7 Medicine & Community Health Sciences, University of Calgary, Calgary; 8 Pediatrics, University of Alberta, Edmonton; 9 Pediatrics, Dalhousie University, Halifax; 10 ICES; 11 Institute of Health Policy, Management and Evaluation; 12 Paediatrics, University of Toronto, Toronto; 13 Pediatrics, University of Ottawa; 14 CHEO Inflammatory Bowel Disease Centre, Division of Gastroenterology, Hepatology and Nutrition, CHEO; 15 CHEO Research Institute, Ottawa; 16 Department of Pediatrics, BC Children's Hospital Research Institute, University of British Columbia, Vancouver; 17 Mount Sinai Hospital Centre for Inflammatory Bowel Disease, Department of Medicine, University of Toronto, Toronto; 18 Pediatrics, University of Manitoba, Winnipeg, Canada

## Abstract

**Background:**

Marked variation in access to care and health services utilization is a marker of variation in quality of care. With the rising incidence of pediatric inflammatory bowel disease (IBD), we must understand variation in access to and outcomes of care to improve quality.

**Purpose:**

Describe variation in care for pediatric IBD treated in 4 Canadian provinces.

**Method:**

Incident cases of IBD diagnosed in children <16y were identified from health administrative data in Alberta (AB), Manitoba, Nova Scotia, and Ontario (ON) using validated algorithms. Children were assigned to one of 8 centres of care using a hierarchical assessment of health services use within 6 months of diagnosis. Children treated by adult gastroenterologists or community-based pediatric gastroenterologists were excluded due to small sample size. Outcomes included IBD-related hospitalizations, emergency department (ED) visits (AB/ON only), and IBD-related abdominal surgery. Hospitalizations and ED visits were counted cumulatively from 6-60 months after diagnosis. The risk of first surgery was defined during the same 6-60 month period. Mixed-effects meta-analysis was used to pool results across centres. Heterogeneity among centres was quantified using I^2^ (variation in pooled event rates between centres) and τ (standard deviation of the true event rates). R^2^ quantified the residual heterogeneity in outcomes not attributable to among-province variation.

**Result(s):**

We identified 3777 incident cases of pediatric IBD, 2936 (78%) of which were treated at 8 pediatric centres. The number of hospitalizations was 0.67 (95% CI 0.56-0.79) per person with high between-centre heterogeneity (I^2^ 84%, τ 0.1556). Provincial differences accounted for 93% of heterogeneity across centres (residual heterogeneity: I^2^ 29%, τ 0.0412). Hospitalizations were less frequent in AB than other provinces (0.43 vs. 0.72-0.78). Children averaged 1.94 IBD-related ED visits, with significant heterogeneity (I^2^ 99%, τ 1.33) with 99.7% of heterogeneity attributable to among-province differences (residual heterogeneity: I^2^ 32%; τ 0.074). Mean ED visits were 1.1 visits in ON (I^2^ 39%) and 3.7 in AB (I^2^ 0%). Intestinal resection was required by 12% (95% CI 0.08-0.15) of Crohn’s patients with high among-centre heterogeneity (I^2^ 81%, τ 0.042), and low (19%) heterogeneity due to provincial differences (residual heterogeneity: I^2^ 76%; τ 0.039). Colectomy was required by 12% (95% CI 10-14) of children with ulcerative colitis (UC) with no between-centre heterogeneity (I^2^ 0%, τ 0).

**Conclusion(s):**

There is a high degree of between-province (but not between-centre, within province) variability in health services utilization among children with IBD. There was significant between-centre variability in surgery rates for Crohn’s, but not colectomy for UC. Differences in patient characteristics or provincial health systems may be more important predictors of variation in care. Surgery for Crohn’s disease may be a target for inter-centre quality improvement efforts.

**Please acknowledge all funding agencies by checking the applicable boxes below:**

CCC

**Disclosure of Interest:**

None Declared

